# The origin of N isotopic discrimination and its relationship with feed efficiency in fattening yearling bulls is diet-dependent

**DOI:** 10.1371/journal.pone.0234344

**Published:** 2020-06-05

**Authors:** Sayyed Mahmoud Nasrollahi, Sarah Jade Meale, Diego P. Morgavi, Anne Marie Schiphorst, Richard J. Robins, Isabelle Ortigues-Marty, Gonzalo Cantalapiedra-Hijar

**Affiliations:** 1 INRAE, Université Clermont Auvergne, Vetagro Sup, UMRH, Saint-Genès-Champanelle, France; 2 School of Agriculture and Food Sciences, The University of Queensland, Gatton Campus, Gatton, Australia; 3 Université de Nantes, CNRS, CEISAM UMR 6230, Nantes, France; Universidade Federal de Viçosa, BRAZIL

## Abstract

Nitrogen (**N**) isotopic discrimination (i.e. the difference in natural ^15^N abundance between the animal proteins and the diet; **Δ**^**15**^**N**) is known to correlate with N use efficiency (**NUE**) and feed conversion efficiency (**FCE**) in ruminants. However, results from the literature are not always consistent across studies, likely due to isotopic discrimination pathways that may differ with the nature of diets. The objective of the present study was to assess at which level, from rumen to tissues, Δ^15^N originates and becomes related to NUE and FCE in fattening yearling bulls when they are fed two contrasted diets. Twenty-four Charolais yearling bulls were randomly divided into two groups and fed during 8 months, from weaning to slaughter, either 1) a high starch diet based on corn silage supplying a balanced N to energy ratio at the rumen level (**starch**) or 2) a high fiber diet based on grass silage supplying an excess of rumen degradable N (**fiber**). All animals were slaughtered and samples of different digestive pools (ruminal, duodenal, ileal and fecal contents), animal tissues (duodenum, liver and muscle), blood and urine were collected for each animal. Ruminal content was further used to isolate liquid-associated bacteria (**LAB**), protozoa and free ammonia, while plasma proteins were obtained from blood. All samples along with feed were analyzed for their N isotopic composition. For both diets, the digestive contribution (i.e. the N isotopic discrimination occurring before absorption) to the Δ^15^N observed in animal tissues accounted for 65 ± 11%, leaving only one third to the contribution of post-absorptive metabolism. Concerning the Δ^15^N in digestive pools, the majority of these changes occurred in the rumen (av. Δ^15^N = 2.12 ± 0.66‰), with only minor ^15^N enrichments thereafter (av. Δ^15^N = 2.24 ± 0.41‰), highlighting the key role of the rumen on N isotopic discrimination. A strong, significant overall relationship (n = 24) between Δ^15^N and FCE or NUE was found when using any post-absorptive metabolic pool (duodenum, liver, or muscle tissues, or plasma proteins; 0.52 < r < 0.73; P ≤ 0.01), probably as these pools reflect both digestive and post-absorptive metabolic phenomena. Fiber diet compared to starch diet had a lower feed efficiency and promoted higher (P ≤ 0.05) Δ^15^N values across all post-absorptive metabolic pools and some digestive pools (ruminal, duodenal, and ileal contents). The within-diet relationship (n = 12) between Δ^15^N and feed efficiency was not as strong and consistent as the overall relationship, with contrasted responses between the two diets for specific pools (diet x pool interaction; P ≤ 0.01). Our results highlight the contrasted use of N at the rumen level between the two experimental diets and suggests the need for different equations to predict FCE or NUE from Δ^15^N according to the type of diet. In conclusion, rumen digestion and associated microbial activity can play an important role on N isotopic discrimination so rumen effect related to diet may interfere with the relationship between Δ^15^N and feed efficiency in fattening yearling bulls.

## Introduction

Improving N use efficiency (**NUE**), the animal’s ability to transform feed N into animal proteins, is a key concern in animal production. However, because this measurement is labor intensive, especially in growing animals where the animal protein accretion is difficult to measure, researchers are searching for easy-to-use and accurate NUE biomarkers. One suggested biomarker of NUE in growing ruminants is based on the natural ^15^N enrichment of animal proteins over the diet [1, 2]. Nitrogen exists in nature as two stable isotopes: the light ^14^N and the far less abundant heavy ^15^N. The natural ^15^N abundance (**δ**^**15**^**N**; ^15^N/^14^N ratio relative to atmospheric N_2_) in animal proteins is higher relative to the diet consumed [3], the so-called N isotopic discrimination between the animal and its diet (**Δ**^**15**^**N** = δ^15^Nanimal—δ^15^N_diet_). A recent meta-analysis found that the more efficiently the ruminant assimilates the dietary N (i.e. high NUE) the closer are the δ^15^N values in animal proteins and diet (i.e. lower Δ^15^N) with, however, a relationship which could be diet-dependent [4]. In particular, diets with high rumen degradable N content seem to interfere with this relationship, possibly due to an as yet unknown rumen effect [4].

The Δ^15^N originates from the isotopic selectivity of metabolic processes (notably enzymes), leading to different isotope ratios between substrates and products [5, 6]. The principle cause of this selectivity is the different strength of the C-^14^N and C-^15^N bonds, which influences the kinetics of the reaction mechanism [7]. Hepatic transaminase and deaminase have been suggested as key factors in N isotopic discrimination because they react more readily with substrates (amino acids) containing ^14^N over ^15^N [5, 7]. As a consequence, the enzymatic isotopic discrimination results in a relatively higher excretion of ^14^N in the end-product (urea) and higher retention of ^15^N in body proteins [6]. As these enzymes are involved in amino acid catabolism and ureagenesis [8], Δ^15^N is biologically linked to NUE. But, microbial enzymes (transaminase and deaminase), such as those present in the rumen, may also discriminate N isotopes during ammonia assimilation into microbial protein [9] or during ammonia release from amino acid catabolism [10]. Variable Δ^15^N values have been reported in single cell organisms ranging from negative [11, 12] to positive [9] values, likely reflecting differences in the balance between N and energy supplied by substrates [11, 13]. In this regard, when the available N is in excess, the resulting increase in rumen ammonia concentration [14] is associated to higher Δ^15^N values in the rumen microbes [12, 15] and in animal proteins [15]. Therefore, microbial activity in the rumen could contribute to the diet-dependent relationship previously found between Δ^15^N and NUE [4].

Direct measurements of Δ^15^N in several digestive (pre-absorption) and metabolic (post-absorption) pools could help unravel the origin of isotopic discrimination, especially when diet composition changes, and its relationship with feed efficiency. The present study was designed as an extensive exploration of natural N isotopic discrimination in different animal body pools of Charolais yearling bulls fed two contrasted diets and its relationship with feed efficiency. We hypothesized that rumen microbial activity contributes to Δ^15^N, and that its contribution increases when there is an imbalance between N and energy at the rumen level, thereby reducing the relationship between Δ^15^N and feed efficiency.

## Material and methods

The experiment was conducted at the animal facilities of INRA UE1414 Herbipôle Unit (Saint-Genès Champanelle, France). All animal procedures were approved by the French Ministry of Education and Research (APAFIS no. 2930–015111814299194) and carried out in accordance with European guidelines and regulations for experimentation with animals.

### Animals and diets

Twenty-four pure-bred Charolais yearling bulls, averaging 390 ± 20 days old and 446 ± 44 kg BW were used in this experiment lasting 8 months. Animals were housed indoors in free stalls. Yearling bulls were offspring of multiparous cows from the same herd sired by 21 different bulls. Individual feed intake was recorded throughout the experiment by automatic intake recording systems based on mangers placed on weighing cells (Biocontrol, Rakkestad, Norway). Yearling bulls were divided into two homogenous groups according to body weight and age and then randomly allocated to two diets: 1) a high starch diet based on corn silage (**starch**) supplying a balanced N to energy ratio at the rumen level and so leading to an estimated rumen protein balance close to 0 (i.e. rumen ammonia absorption equals recycled urea into the rumen) or 2) a high fiber diet based on grass silage (**fiber**) supplying rumen degradable N in excess and so leading to a highly positive rumen protein balance (i.e. higher rumen ammonia absorption compared to the recycled urea into the rumen). The two diets contained around 65% of forage and 35% of concentrate and were distributed once a day (8:00 h) as TMR *ad libitum* with offering adjusted daily to ensure around 100 g/kg refusals. The ingredients, chemical composition, and feeding values of the diets are presented in Table 1. Fiber diet was formulated mainly by using high fiber ingredients (grass silage and beet pulp) while starch diet was formulated mainly by including high starch ingredients (corn silage and wheat grain). Soybean meal was included to give diets a similar crude protein (CP) content. Diets were formulated to differ in carbohydrate composition and rumen protein balance while still promoting good performances and avoiding health problems related to rumen function. Compared to the starch diet, the fiber diet contained greater neutral detergent fiber (**NDF**; 43.6% vs 30.5%) and rumen degradable protein (75.4% vs 65.0%) while much lower starch (5.65% vs. 42.1%). The estimated dietary net energy for growth and maintenance was lower for fiber vs starch diets (1.36 Mcal/kg DM vs 1.57 Mcal/kg DM) and thus leading to lower estimated average daily gain (**ADG**; 1450 g/d vs 1650 g/d) [16]. Although the net energy and metabolizable protein (**MP**) content differed across the two diets, the ratio between both remained relatively close and above recommendations [16]. Feeds were sampled three times per week throughout the experiment. Feeds were ashed at 550 °C for 6 h for organic matter (OM) determination. The nitrogen content of feeds was determined by the Kjeldahl procedure [17], the NDF and acid detergent fiber (ADF) were determined according to Van Soest et al. [18] and starch in the concentrate was determined by spectrophotometry after enzymatic analysis [19].

**Table 1:** Ingredient and chemical composition of experimental diets tested on fattening Charolais yearling bulls.

Item	Fiber diet	Starch diet
**Ingredient composition, % of DM**		
**Forage**		
**Corn silage**		60
**Grass silage**	60	
**Wheat straw**	5	5
**Concentrate**		
**Wheat grain**	6.6	22.7
**Beet pulp**	26.3	
**Soybean meal**	2.1	12.3
**Chemical composition, %DM**		
**OM**	88.6	95.0
**CP**	14.0	14.4
**NDF**	43.6	30.5
**ADF**	23.6	13.8
**Starch**	5.1	42.1
**Estimated feed values**^a^^b^		
**Rumen degradable protein**^c^**, %**	75.4	65.0
**RPB**^d^**, g/kg DM**	10.1	1.46
**Net energy**^e^**, Mcal/kg DM**	1.36	1.57
**PDIA**^f^**, g/kg DM**	19	36
**PDI**^g^**, g/kg DM**	71	86
**PDI/NEg**^h^	51.9	54.8

^a^Chemical composition and feed values correspond to feed samples pooled throughout the experimental period

^b^Feed values were estimated from chemical composition and INRA equations [16] using Systool web software 1.2

^c^Rumen degradable protein estimated from measured enzymatic CP degradability (concentrates) and chemical composition (forages)

^d^Rumen protein balance = CP content of diets minus non-NH_3_ CP concentration at duodenum

^e^Net energy value for maintenance and growth in fattening yearling bulls

^f^PDIA = rumen by-pass protein digestible in the small intestine

^g^PDI = protein digestible in the small intestine (equivalent to metabolisable protein)

^h^Metabolizable protein (PDI) to Net Energy ratio. Recommended values are around 51 for beef cattle of 400 kg and 48 for beef cattle of 600 kg [16].

For estimating the effective degradability of N the enzymatic CP degradability of concentrates was assessed by *in vitro* protease hydrolysis for 1 h according to Aufrère et al. [20]. The net energy and metabolizable protein (PDI) values of the diets (Table 1) were calculated according to the INRA feeding system [16] using the Systool Web software (www.systool.fr) taking into account the average BW and DM intake observed during the study (and determining the extent of digestive interactions along with the concentrate percentage) and the measured chemical composition of each feed ingredient. For MP content calculation, the effective degradability of N (ED) of concentrate ingredients was derived from their measured *in vitro* CP digestibility, and their theoretical true intestinal digestibility from tabulated values [16]. For silages, both ED and the true intestinal digestibility were derived from their chemical composition, according to INRA [16].

### In vivo measurements and sampling

Body weight (BW, kg) was determined every 14 d at 14:00 h without a previous feed restriction (6 h after meal distribution) throughout the experiment, with two records obtained in two consecutive days for the beginning and end of the experiment. At the onset of the experiment all animals were biopsied under local anesthesia from the middle of a triangle formed by the last lumbar vertebrae, tail base and ischial tuberosity. Subcutaneous adipose tissue was sampled to determine the diameter of adipocytes and thus to estimate the fat mass in the empty body weight at the beginning of the trial according to equations developed by Garcia and Agabriel [21] and previously detailed [2]. On the day preceding the slaughter of each animal, blood was sampled at 8:00 h before feeding by venipuncture from the jugular vein using 9 mL evacuated tubes with lithium heparin as an anticoagulant (BD vacutainer, Plymouth, UK). Tubes containing the blood were centrifuged at 2,500 × *g* for 15 min at 4°C and stored at −20°C before plasma protein isolation and N isotopic analysis.

### Post-mortem measurements and sampling

Slaughters were carried out once the first four animals reached a body weight of around 720–740 kg (corresponding to a target market carcass weight of around 420 kg). As the sampling protocol was labor intensive, animals were slaughtered over a 6 week period at the rate of four animals per week at the INRA’s experimental slaughterhouse of UE1414 Herbipôle Unit. Two animals for each dietary treatment were slaughtered on the same day to keep the same fattening length across dietary treatments, so the average age at slaughter was the same for the two dietary treatments. Internal fat (heart, mesenteric, pelvic, and kidney), and visceral organ weights were recorded. For all animals, the sixth rib was dissected to estimate the weight of bone, muscle, and fat in whole body (in kg) according to the equations described by Robelin and Geay [22] for Charolais yearling bulls.

Ruminal contents (around 2 kg) were collected at slaughter from different parts of the rumen and pooled to produce a homogenous sample representing the whole-rumen content. Then, they were strained through a 250 μm nylon monofilament filter to separate particles from the liquid. The solid part was mixed with 1 L of pre-warmed (39°C) saline solution (0.9%) and filtrated again through the nylon filter. This second filtrate was pooled with that obtained in the first separation and then split equally into two identical flasks, each one used for isolation of liquid associated bacteria (**LAB**) and protozoa. For LAB we followed the procedure described in [23] where the liquid content was first centrifuged at 1,000 × *g* during 10 min at 4°C. The decanted supernatant was then centrifuged at 27,000 × *g* during 25 min at 4°C. The resulting microbial pellet was rinsed with saline solution (0.9%) and then kept at -20°C until isotopic analysis. The supernatant obtained after the second centrifugation was immediately used (5 mL) for ammonia isolation through a microdiffusion technique [24] that uses potassium carbonate in a first compartment to favor ammonia volatilization from the supernatant and temperature and boric acid to trap it in a second compartment (3 h at room temperature). Protozoa isolation was done using the protocol described by Martin et al. [25]. The isolation was performed on the second flask that was kept under anaerobic conditions and at 39°C throughout the procedure. Briefly, a small amount of glucose (1g/L) was added into the filtrate to stimulate flocculation of small feed particles and favor sedimentation of protozoa. A minimum of 10 min after the introduction of glucose and when a scum layer of feed particle was formed, floating particles were removed by a vacuum suction system and the remaining liquid centrifuged at 1,000 × *g* during 10 min. The protozoal pellet was washed on a 20 μm nylon filter with saline solution and stored in 15 mL falcon tubes at -20°C before isotopic analysis.

Digesta contents from the intestines were sampled from 30 cm of each section. Proximal duodenum was clamped and cut at 5 and 35 cm caudal from the pyloric sphincter. Terminal ileum was clampled and cut at 30 cm before the beginning of the cecum. Finally, fecal material was sampled from the last section of the rectum, before the anorectal junction. Animal tissues were sampled within the first 30 min after slaughter. Around 300g of tissue from the proximal duodenum, *longissimus dorsi* muscle and hepatic caudal lobe were sampled and kept at -20°C. To avoid any contamination with the digesta content, duodenal tissue was rinsed twice with saline (0.9%) during 1 min each.

### Isotopic analysis

The analysis of natural relative abundance of the rare stable isotope of N (δ^15^N) was carried out as described previously [2]. Samples from all animal pools (except urine, rumen ammonia and plasma proteins) and feed were freeze-dried and ground to pass a 1-mm sieve. Upon thawing at 4°C overnight, the protein fraction from plasma was isolated by precipitation with sulfosalicylic acid (37.5 μl of a 1g/ml solution into 0.75 ml of sample). After 1-h storage at 4 °C and centrifugation (5000 × *g* for 15 min at 4°C), the supernatant was discarded and the pellet was rinsed three times with milliQ water (Millipore, Bedford, MA, USA), before being freeze-dried. For liquid samples (urinary and rumen ammonia) around 14 mg were introduced into a silvered cap (3.5 × 5.5 mm) and evaporated at 25–30°C during 1 night under an inert gas flow. The remaining dry matter was analyzed once the cap was folded. Nitrogen stable isotopic composition, δ^15^N (‰) of each body pool and diets were determined by isotope ratio measuring mass spectrometry (irm-MS) using an Integra 2 continuous flow irm-MS (Sercon Ltd., Cheshire, UK) following total combustion in an elemental analyzer (Sercon Ltd., Cheshire, UK). Results were expressed using the delta notation in parts per 1000 (‰) in relation to the international standards, atmospheric N2 (R_standard_ = 0.0036765), via the in-house standard glutamic acid (run every ten samples).

### Calculations

Feed efficiency was measured during the overall experimental period (240 days) to calculate feed conversion efficiency (**FCE**) for each animal as the ADG divided by the daily dry matter intake. The ADG for each animal was determined as the coefficient of the linear regression of BW on time (days). MP use efficiency was calculated as the estimated N retained in the animal body divided by the MP intake.

Nitrogen use efficiency was calculated based on whole-body nitrogen retention divided by nitrogen intake as described in detail in Cantalapiedra-Hijar et al. [2]. In brief, whole-body nitrogen retention was estimated from the difference between body nitrogen at the start and the end of experiment. The total amount of body proteins at the onset of the experiment was estimated from adipocyte size measured at this time, while total amount of body protein at the end of experiment were estimated from carcass composition that calculated from the 6^th^ rib dissection method [22].

The contribution (%) of digestion to the Δ^15^N values observed in animal tissues was calculated as the average of Δ^15^N values from duodenal and ileal contents (the anatomical site where amino acids are digested and absorbed) divided by the average of Δ^15^N values from animal tissues (muscle, liver, duodenum and plasma). The contribution (%) of post-absorptive metabolism to the Δ^15^N values observed in animal tissues was calculated as 100 –the digestive contribution.

### Statistical analyses

All statistical analyses were performed using R software (version 3·5·1). We considered repeated measurement from the same experimental unit across anatomical pools. Thus a mixed effect model was built for explaining the Δ^15^N values across diets and pools according to the following model:

$${\text{Y}}_{\text{ijkl}}=\text{mean}+{\text{diet}}_{\text{i}}+{\text{pool}}_{\text{j}}+{\left(\text{pool}\times \text{diet}\right)}_{\text{ij}}+{\text{animal}}_{\text{ijk}}+{\text{e}}_{\text{ijkl}}$$ (1)

In this model, diet was the fixed effect of diet (i = 2), pool was the fixed effect of pool (j = 11), and animal was the random effect of animal (k = 24) and e_ijkl_ was the random residual error. Anova was performed when comparisons between diets were conducted within each body pool. Least squares means were presented, and significant differences or trends were declared at P ≤ 0.05 and P ≤ 0.10, respectively. The means were compared using the pairwise *t*-test option after a significant (*P* ≤ 0.05) overall *F*-test. The correlation coefficients between variables were determined using cor.test function in R (version 3·5·1).

## Results

### Animal performance

**Fig 1:**
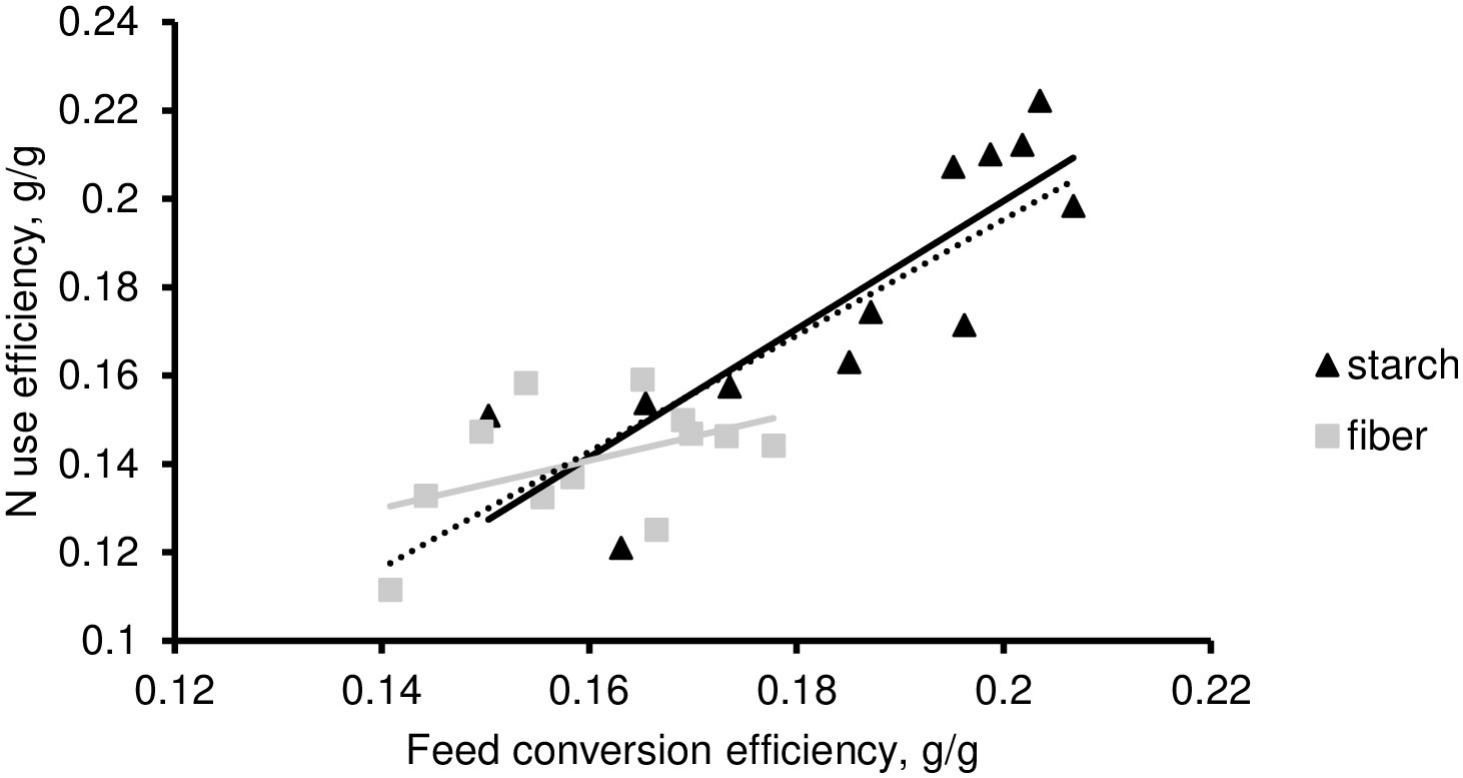
The relationship between N use efficiency (NUE) and feed conversion efficiency (FCE) in fattening Charolais yearling bulls fed either fiber or starch diets. The dashed line is the overall relationship. Equations for the overall, within-starch and within-fiber relationships are NUE = 1.314×FCE– 0.067 (r^2^ = 0.74; P < 0.01), NUE = 1.450×FCE– 0.091 (r^2^ = 0.73; P < 0.01), and NUE = 0.530×FCE + 0.055 (r^2^ = 0.21; P = 0.13), respectively.

While average dry mater and N intake were similar for both diets, the ADG and the amount of retained N were greater for the starch compared to the fiber diet (Table 2). Consequently, FCE and NUE were also greater (P < 0.01) with the starch diet (0.186 and 0.179, respectively) compared to the fiber diet (0.160 and 0.141, respectively). The overall relationship between FCE and NUE was high (r^2^ = 0.74; P < 0.01). This relationship was mostly driven by results obtained with the starch diet, as when diets were considered separately the within-diet relationship was similar to the overall one with the starch diet (r^2^ = 0.73; P < 0.01), but not with the fiber diet (r^2^ = 0.21; P = 0.13; Fig 1). Calculated MP use efficiency was numerically higher for starch (0.310) vs fiber diets (0.284) but not significantly different (P = 0.14).

**Table 2:** Effect of experimental diets (fiber vs starch diets) on performances in fattening Charolais yearling bulls.

Item	Fiber diet	Starch diet	SE	P-value
**DM Intake, kg/d**	9.46	9.74	0.434	0.52
**ADG, kg/d**	1.52	1.81	0.102	0.01
**FCE, g/g**	0.160	0.186	0.004	<0.01
**N intake**	0.216	0.233	0.007	0.11
**Retained N**^a^**, kg/d**	0.031	0.042	0.002	<0.01
**NUE, g/g**	0.141	0.179	0.007	<0.01
**MP use efficiency**^b^**, g/g**	0.284	0.310	0.012	0.14

^a^Retained N was estimated from the sixth rib dissection and subcutaneous adipose tissues data as detailed by Cantalapiedra-Hijar et al. [2]

^b^MP use efficiency was calculated as retained N divided by MP intake (PDI intake)

### Isotopic N discrimination across diets and body pools

The change of Δ^15^N across body pools differed between diets (interaction Δ^15^N×pool; P < 0.01; Table 3). To facilitate the clarity of the results, we report the effect of diet on Δ^15^N within each body pool (within row comparison in Table 3) and the effect of the body pool on Δ^15^N within each diet (within column comparisons in Table 3).

**Table 3:** Effect of diet and body pool on N isotopic discrimination (Δ^15^N; ‰) in fattening Charolais yearling bulls.

	Fiber diet	Starch diet	P-value^a^
**Digestive pools**			
**Ruminal content**	2.36^B^	1.79^D^	0.01
**Rumen LAB**	1.37^C^	1.49^D^	0.58
**Rumen Protozoa**	2.87^B^	3.04^A^^B^	0.36
**Duodenal content**	2.24^B^	1.81^C^^D^	0.08
**Ileal content**	2.62^B^	2.27^C^	<0.01
**Faeces**	2.62^B^	2.73^B^	0.45
**Post-absorptive metabolic pools**			
**Plasma proteins**	4.04^A^	3.19^A^	<0.01
**Duodenal tissue**	3.58^A^	2.83^B^	<0.01
**Liver**	3.96^A^	3.37^A^	<0.01
**Muscle**	3.92^A^	2.97^B^	<0.01
**Catabolic end-products**			
**Rumen ammonia**	-1.01^D^	-1.13^E^	0.11
**Urine**	-1.74^E^	-2.35^F^	<0.01
**SE**	0.147		
**P value (diet)**	<0.01		
**P value (pool)**	<0.01		
**P value (pool×diet)**	<0.01		
**Digestive pools**^b^	2.43	2.05	0.02
**Post-absorptive metabolic pools**^c^	3.87	3.09	<0.01

^A-F^Least squares means within a column with different superscripts differ significantly (*P* ≤ 0.05).

^a^ P-value from the mean comparison within a row (i. e. test on diet effect within each pool).

^b^Δ^15^N average values from duodenal and ileal contents (SE = 0.159 ‰).

^c^Δ^15^N average values from plasma proteins, duodenal tissue, liver and muscle (SE = 0.099‰).

#### Effect of diet on Δ^15^N

The Δ^15^N values in the ruminal, duodenal, and ileal contents were greater (P ≤ 0.08) in animals fed fiber diet (av. 2.41‰) compared to those fed starch diet (av. 1.96‰). On the other hand, Δ^15^N values remained similar across the two diets (P > 0.10) in rumen LAB, protozoa, and fecal samples. The Δ^15^N values in duodenal tissue, plasma proteins, liver, and muscle were always greater (av. +0.76 ‰; P < 0.01) in fiber vs. starch diets. Likewise, Δ^15^N values in urine, despite being negative (depletion compared to diets) were greater in fiber vs. starch diets (-1.74 vs. -2.35‰; P < 0.01), whereas Δ^15^N in rumen ammonia was only numerically greater (-1.01 vs. -1.13; P = 0.11).

#### Effect of pools on Δ^15^N

For the fiber diet, urine had the lowest Δ^15^N value, then rumen ammonia and thereafter LAB. With this diet, most samples from the digestive pools (ruminal, duodenal, and ileal contents, rumen protozoa, and feces) did not show differences in Δ^15^N values. Furthermore, although Δ^15^N values from the post-absorptive metabolic pools (plasma proteins, duodenal tissue, liver, and muscle) were all similar, they were higher than those from the digestive pools (P < 0.01). For the starch diet, however, the change of Δ^15^N values across body pools followed a different pattern. Although urine and ammonia were the first and second pools with the lowest Δ^15^N values, as observed for the fiber diet, the Δ^15^N values in ruminal and duodenal contents were lower than that observed in the ileal content (P < 0.05), which was in turn lower than feces (P < 0.05). Duodenum and muscle had isotopic signatures comparable to feces (P > 0.05), but they had lower Δ^15^N values than plasma proteins and liver (P < 0.05). The Δ^15^N value in rumen protozoa was not significantly different from those observed in body tissues (P > 0.05).

**Fig 2:**
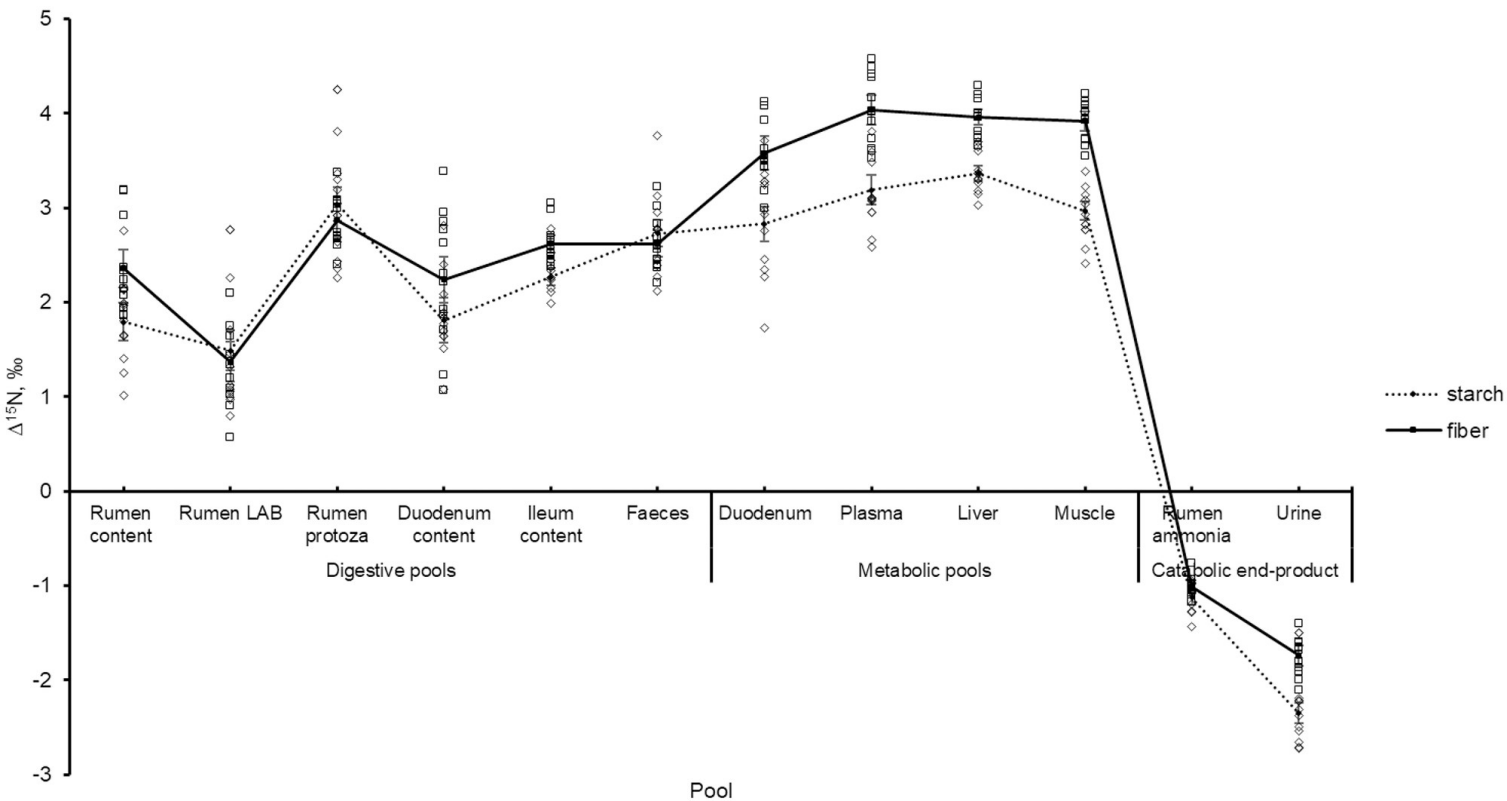
Nitrogen isotopic discrimination (Δ^15^N) across different animal body pools in fattening Charolais yearling bulls fed either fiber or starch diets. The open squares and diamonds are individual values for fiber and starch diets, respectively. Samples are grouped according to digestive, metabolic, and catabolic end-product pools.

The change of Δ^15^N across pools are illustrated in Fig 2, with the values for each individual animal plotted around each treatment mean. All of the digestive and post-absorptive metabolic pools had a positive value of Δ^15^N, indicating that that these pools were isotopically enriched relative to the diet. Overall, Δ^15^N values measured in the digestive pools were lower than those from the post-absorptive metabolic pools (P < 0.01). The pools representing end-products from N catabolism (rumen ammonia and urine) had negative Δ^15^N values, indicating that they were isotopically depleted in ^15^N compared to the diet.

### Relationship between Δ^15^N and feed (N) efficiency

The overall relationship (n = 24) between Δ^15^N and FCE in almost all pools were negative, but correlation coefficients varied in strength and significance (Table 4). Regarding digestive pools, the Δ^15^N values in both ileal content (r = -0.48) and rumen LAB (r = -0.41) were significantly correlated to FCE (P < 0.05). The relationship between FCE and Δ^15^N in post-absorptive metabolic pools were strongly significant (P < 0.001) with correlation coefficients (r) ranging from -0.51 to -0.69. The Δ^15^N values in both rumen ammonia and urine were correlated to FCE with a correlation coefficient of -0.52 and -0.73, respectively (P < 0.01). Within-diet relationship between Δ^15^N and FCE, however, were less consistent and with weaker correlation coefficient compared to the overall relationship. Significant or a trend for correlations were only noted for rumen ammonia and muscle pools (P ≤ 0.03) in fiber diets, and for LAB and urine pools (P ≤ 0.07) in starch diets.

**Table 4:** Overall and within-diet relationship between feed efficiency and N isotopic discrimination (Δ^15^N) in fattening Charolais yearling bulls fed either fiber or starch diets^1^.

	Overall			Fiber diet			Starch diet		
	r	se	P value	r	se	P value	r	se	P value
**Digestive pools**									
**Ruminal content**	-0.26	0.206	0.22	0.09	0.315	0.78	0.18	0.311	0.58
**Rumen LAB**	-0.41	0.195	0.05	-0.20	0.309	0.51	-0.84	0.171	<0.001
**Rumen protozoa**	-0.12	0.212	0.58	-0.35	0.296	0.26	-0.31	0.300	0.32
**Duodenal content**	-0.29	0.204	0.17	-0.29	0.305	0.41	0.06	0.315	0.83
**Ileal content**	-0.48	0195	0.02	-0.31	0.301	0.33	0.02	0353	0.96
**Faeces**	0.05	0.213	0.81	-0.42	0.288	0.18	0.08	0.315	0.80
**Post-absorptive metabolic pools**									
**Plasma proteins**	-0.69	0.155	<0.001	-0.31	0.301	0.33	-0.45	0.281	0.14
**Duodenal tissue**	-0.51	0.183	0.01	0.17	0.312	0.60	-0.26	0.305	0.40
**Liver**	-0.65	0.161	<0.001	-0.27	0.304	0.38	-0.26	0.305	0.40
**Muscle**	-0.67	0.158	<0.001	-0.63	0.244	0.03	-0.12	0.313	0.70
**Catabolic end-products**									
**Rumen ammonia**	-0.52	0.182	0.009	-0.67	0.234	0.02	-0.23	0.307	0.46
**Urine**	-0.73	0.146	<0.001	-0.32	0.299	0.31	-0.54	0.266	0.07

^1^Mean values for feed efficiency and Δ^15^N of the animal pools are reported by diet in Tables 2 and 3, respectively.

The overall relationships between Δ^15^N and NUE were similar to the relationship between Δ^15^N and FCE, but with a slightly lower fitting (Table 5). Likewise, the within-diet relationships were less consistent and weaker than the overall relationship (Table 5) and only significant relationships were noted in the starch diet with plasma proteins and liquid-associated bacteria pools (P ≤ 0.02).

**Table 5:** Overall and within-diet relationship between N use efficiency and N isotopic discrimination (Δ^15^N) in fattening Charolais yearling bulls fed either fiber or starch diets.

	Overall			Fiber diet			Starch diet		
	r	Se	P value	r	se	P value	r	se	P value
**Digestive pools**									
**Ruminal content**	-0.32	0.201	0.11	-0.15	0.313	0.65	0.09	0.315	0.76
**Rumen LAB**	-0.39	0.196	0.06	-0.25	0.306	0.42	-0.74	0.212	0.006
**Rumen protozoa**	-0.10	0.212	0.61	0.02	0.316	0.95	-0.35	0.295	0.25
**Duodenal content**	-0.35	0.220	0.09	-0.46	0.281	0.13	-0.04	0.316	0.91
**Ileal content**	-0.57	0.183	0.005	-0.51	0.272	0.09	-0.19	0347	0.60
**Faeces**	0.05	0.213	0.81	-0.43	0.284	0.16	0.04	0.316	0.88
**Post-absorptive metabolic pools**									
**Plasma proteins**	-0.68	0.156	<0.001	0.17	0.312	0.60	-0.66	0.238	0.02
**Duodenal tissue**	-0.60	0.170	0.002	0.04	0.316	0.89	-0.43	0.285	0.16
**Liver**	-0.70	0.152	<0.001	-0.29	0.302	0.35	-0.47	0.277	0.12
**Muscle**	-0.66	0.159	<0.001	0.02	0.316	0.96	-0.39	0.291	0.20
**Catabolic end-products**									
**Rumen ammonia**	-0.41	0.195	0.05	-0.50	0.273	0.10	-0.12	0.314	0.70
**Urine**	-0.60	0.169	0.002	-0.14	0.313	0.66	-0.29	0.303	0.36

^1^The relative values for feed efficiency and Δ15N of animals within each diet are reported in Tables 2 and 3, respectively.

**Fig 3:**
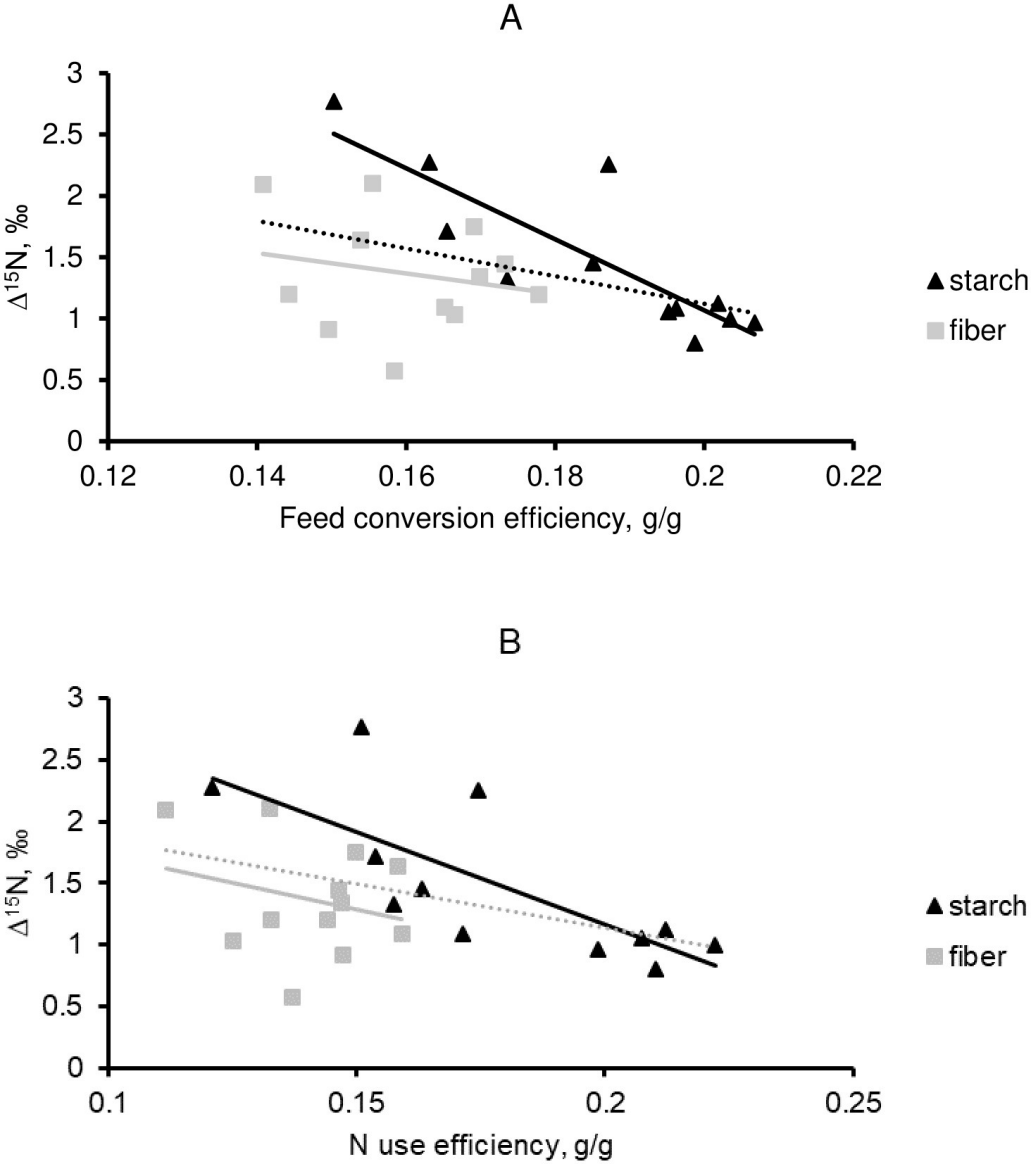
The relationship between Δ^15^N measured in liquid associated bacteria and either feed conversion efficiency (FCE; Panel A) or N use efficiency (NUE; Panel B) in fattening Charolais yearling bulls fed either fiber or starch diets. The dashed line is the overall relationship. For FCE, equations for the overall, within-starch and within-fiber relationships are Δ^15^N = -11.27×FCE + 3.37 (r^2^ = 0.17; P = 0.05), Δ^15^N = -28.97×FCE + 6.86 (r^2^ = 0.71; P < 0.01) and Δ^15^N = -8.29×FCE + 2.69 (r^2^ = 0.04; P = 0.51), respectively. For NUE, equations for the overall, within-starch and within-fiber relationships are Δ^15^N = -7.12×NUE + 2.56 (r^2^ = 0.16; P = 0.06), Δ^15^N = -15.05×NUE + 4.17 (r^2^ = 0.54; P = 0.01) and Δ^15^N = -8.49×NUE + 2.56 (r^2^ = 0.06; P = 0.43), respectively.

**Fig 4:**
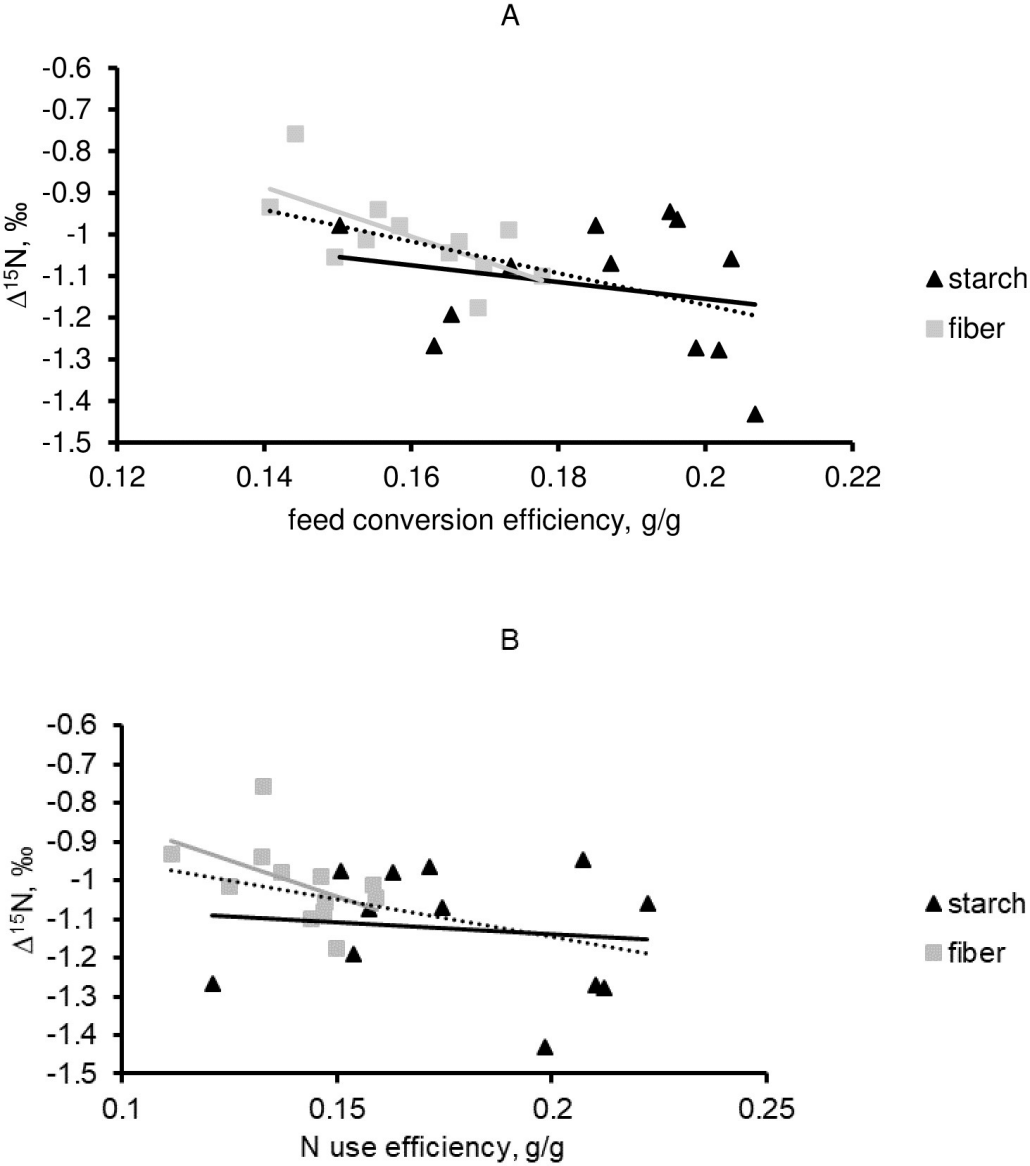
The relationship between Δ^15^N measured in rumen ammonia and either feed conversion efficiency (FCE; Panel A) or N use efficiency (NUE; Panel B) in fattening Charolais yearling bulls fed either fiber or starch diets. The dashed line is overall relationship. For FCE, equations for the overall, within-starch and within-fiber relationships are Δ^15^N = -3.80×FCE—0.41 (r^2^ = 0.28; P = 0.01), Δ^15^N = -2.01×FCE—0.75 (r^2^ = 0.05; P = 0.46) and Δ^15^N = -5.94×FCE—0.06 (r^2^ = 0.45; P = 0.02), respectively. For NUE, equations for the overall, within-starch and within-fiber relationships are Δ^15^N = -1.94×NUE—0.76 (r^2^ = 0.17; P = 0.05), Δ^15^N = -0.63×NUE—1.01 (r^2^ = 0.02; P = 0.70) and Δ^15^N = -3.77×NUE—0.48 (r^2^ = 0.25; P = 0.09), respectively.

As mentioned before, the relationship between Δ^15^N values and feed (N) efficiency was not consistent across diets for some specific body pools. The clearest inconsistency was observed in rumen LAB and rumen ammonia pools (Figs 3 and 4). Indeed, Δ^15^N values in LAB was strongly related to FCE (r^2^ = 0.71; P<0.01) and NUE (r^2^ = 0.54; P = 0.01) but only in animals fed the starch diet, leading to an overall weak correlation. Interestingly, the correlation between Δ^15^N in rumen LAB and FCE within the starch diet was even greater than the relationship from any post-absorptive metabolic pools (Tables 4 and 5). On the other side, when this relationship was regarded in the rumen ammonia pool only a significant correlation was noted for the fiber diet [r^2^ = 0.45 (P = 0.02) for FCE and r^2^ = 0.25 (P = 0.09) for NUE]. Similar to rumen LAB, the relationship obtained with Δ^15^N measured in rumen ammonia was greater with FCE than with NUE.

## Discussion

As NUE is a key component of FCE [1, 2], evidenced here through the strong relationship between both traits (especially with starch diets; Fig 1), it is assumed that proxies for the former should work for the latter. Overall, results from the present study confirmed that Δ^15^N can be proposed as a biomarker of feed efficiency in fattening yearling bulls [1, 2]. Moreover, our results highlight the considerable contribution of rumen function to the N isotopic discrimination in fattening yearling bulls and provides some evidence, further discussed, on how dietary factors (namely the rumen protein balance and the nature of the energy supply) can strongly impact N isotopic discrimination and its relationship with FCE and NUE.

### Isotopic N discrimination

#### Digestion vs metabolism

To evaluate the contribution of different biological processes to the origin of Δ^15^N, all animal pools should be in isotopic equilibrium with the consumed diet. The extended period (eight months) on which bulls were fed the same diet ensured that all animal pools here were sampled in isotopic equilibrium with the consumed diet, even those with very slow turnover rates, such as muscle in beef cattle [26]. In the present study, the Δ^15^N values before absorption (estimated as the average Δ^15^N of duodenal and ileal contents) was 2.2‰ on average for both diets. The digestive contribution (Δ^15^N values before absorption) to the final Δ^15^N in animal tissues (plasma proteins, duodenum, liver and muscle averaged 3.5‰) accounted for 65% (i.e. 2.2/3.5), leaving only one third to attribute to post-absorptive metabolism. Previously, our results with lactating dairy cows suggested a much lower contribution of digestive processes (around 20%) to the final Δ^15^N measured in animal proteins [15]. As isotopic discrimination is a time-dependent process [9, 13], discrepancies between the present beef cattle study and our previous dairy cattle study [15] could stem from a different rumen retention time between these two different conditions. Indeed lactating dairy cows of our previous study had a considerably greater intake than Charolais yearling bulls of the present study (~20 vs. ~10 kg/d or 3 vs. 1.7% of body weight) which can result in a greater passage rate and lower rumen retention time [16]. Another hypothesis could be related to the differences in the type of diets used in both studies. Diets formulated in the present experiment would promote a relatively low digestive efficiency of N utilization (50–60% of total crude protein would theoretically lead to metabolizable protein [PDI]) compared to our previous work (70–80%; [15]).

Concerning the digestive pools, the average value of Δ^15^N in the rumen was 2.1‰ with only minor changes thereafter until absorption, highlighting the key participation of the rumen in the origin of the N isotopic discrimination. Rumen microbial activity is known to play an important role in N isotopic fractionation [12, 15, 27] as confirmed by our Δ^15^N values measured in rumen protozoa (~3‰) and bacteria (~1.4‰) indicating a high natural ^15^N enrichment over the diet. Since microbial protein is the major source of absorbed AA (around 66% of the total N flowing to duodenum in the present study according to theoretical feeding values [16]) the Δ^15^N of rumen microbiota can contribute largely to the final Δ^15^N of animal body proteins. The Δ^15^N values for protozoa were as high as those measured in animal tissues (3.0 vs. 3.5‰ on average) in agreement with a previous study [27] and confirm the natural ^15^N enrichment of organisms (protozoa) over their diets (bacteria) [3]. Protozoal biomass may account for half of total rumen microbial biomass [28] and may potentially have a large contribution to the N isotopic discrimination noted in the ruminal content.

#### Starch vs fiber diets

The balance between rumen degradable N and available energy at the rumen level by feeding a starch vs. a fiber diet had a remarkable impact on the Δ^15^N measured in digestive pools, where higher values were observed in the ruminal, duodenal, and ileal contents of bulls fed the fiber diet which had an excess of rumen degradable N over energy supply (i.e. high rumen protein balance). Results obtained *in vitro* with soil fungi, having the ability to use ammonia as N source like rumen bacteria, showed that a high N to energy ratio in the medium significantly increased the Δ^15^N [13]. These results support the hypothesis that Δ^15^N could be used as an internal marker to trace N metabolism of microorganisms, as was found *in vitro* with rumen bacteria [12]. Surprisingly, however, the N isotopic discrimination in rumen LAB and protozoa pools were not affected by diet in the current study. Although the underlying mechanism is not completely understood, it could potentially be related to the activity of solid associated bacteria (**SAB**), not isolated in our study due to technical problems. Greater Δ^15^N in SAB have been observed with diets promoting a greater availability of N over energy at the rumen level [15]. The possible high rumen ammonia production promoted by the fiber diet, suggested by the high rumen protein balance values (Table 1), together with a higher NDF content are suitable conditions for the activity and growth of cellulolytic bacteria that predominate in the SAB pool [29, 30]. The second possibility for greater Δ^15^N value in digestive pools with fiber vs starch diet might be related to endogenous secretions, which may contribute ~13% of the total N flowing to the duodenum [31]. Indeed, as reported in the present study all body tissue and blood plasma proteins were naturally ^15^N enriched over the diet and therefore, tissue desquamation and metabolic secretions from digestive surfaces could indirectly increase the value of Δ^15^N in digestive pools. This hypothesis may suggest an overestimation on Δ^15^N in the digestive pools because part of N isotopic discrimination may not originate directly from digestion, but from metabolism.

Similar to the digestive pools, greater Δ^15^N values were found in all metabolic pools of animals fed the fiber diet. Although such a difference could be the consequence of differences observed in the digestive pools, it was still significant when true metabolic Δ^15^N values were calculated by subtracting the Δ^15^N values before absorption (1.44 vs 1.04‰ for fiber vs. starch diet; P = 0.02). This finding supports the role of post-absorptive metabolism in N isotopic discrimination previously observed in ruminants [1, 15] that might be related to the liver transamination and deamination reactions [1, 32]. In this regard, high starch diets promote a higher absorption of glucogenic substrates, which in turn can spare amino acids from catabolism [33, 34]. Lower amino acid catabolism would be expected in starch vs fiber diets and thereby, lower N isotopic discrimination. In this sense, our estimated MP use efficiency supports a better use of amino acids with starch (0.310) vs fiber (0.284) diets, despite being non-significant.

#### Relationship of N isotopic discrimination with FCE and NUE

Our results confirm previous findings showing a significant, negative relationship between Δ^15^N and FCE or NUE [1, 35] with a stronger relationship when Δ^15^N is measured at the metabolic vs digestive level [15]. It should be noted that variation in Δ^15^N in animal tissues reflect both digestive (before absorption) and metabolic (post-absorption) mechanisms related to N isotopic fractionation. In this regard, when Δ^15^N in metabolic pools were adjusted by subtracting values observed before absorption, then the relationship between Δ^15^N and overall FCE or NUE became logically much weaker (r = -0.36 and P = 0.08 for FCE and r = -0.39 and P = 0.06 for NUE). Therefore, the strong relationship observed with FCE or NUE when Δ^15^N measured in animal tissues should be interpreted as reflecting what happens both at the digestive and metabolic levels.

To be considered as a promising biomarker at the individual level, N isotopic discrimination should be able to detect between-animal variation in feed efficiency across different dietary conditions. The within-diet relationships, closely related to between-individual variability, were weaker than those obtained at the overall level and most pools did not show a significant relationship between Δ^15^N and FCE or NUE. This finding could be related to the low number of animal used within each diet (n = 12) and higher experimental errors associated with the between-animal variation. It seems that part of the strength of the overall relationship was due to different intercepts (mean values) across diets (Table 2), which reflect dietary effects rather than between-animal variations. In addition, the range of between animal variation for NUE and FCE was relatively low within-diet (especially for the fiber diet) and insufficient to reach a significant relationship with Δ^15^N in all pools. Still, it should be highlighted that some within-diet relationship keep a relatively good fitting (r < -0.4) and some like those observed for plasma proteins, muscle, rumen LAB and rumen ammonia, were significant.

The dependency of the relationship between Δ^15^N and FCE or NUE on diet was mainly due to two specific pools: rumen bacteria and rumen ammonia. The Δ^15^N measured in LAB significantly correlates with FCE (r2 = 0.70) or NUE (r2 = 0.54) but only when animals were fed the starch diet, and the correlation was stronger than with any other metabolic pool. By contrast, Δ^15^N measured in rumen ammonia only correlated with FCE or NUE in animals fed the fiber diets. This finding highlights the contrasted use of N at the rumen level between the two diets. The greater amount of rumen fermentable organic matter in the form of starch would be mainly used as an energy source for starch-degrading bacteria dominantly located in the LAB pool [36]. In contrast, the fiber diet contained more substrate favoring SAB growth [36, 37] which use ammonia as the main N source [33]. Although speculative, this suggests that individual variability in NUE at the rumen level is mostly driven by rumen LAB metabolism when animals are fed diets containing high amounts of starch, whereas with high-cellulose diets promoting high rumen ammonia concentration, a higher contribution from SAB metabolism is expected. Further studies are warranted to confirm these results and the underlying hypothesis.

As N isotopic discrimination occurs in several metabolic pathways dealing with N at different levels [5, 7], it was expected that Δ^15^N is more correlated to NUE than to FCE. However, an opposite trend has been found in this study. A plausible reason could be that the method to estimate N retention in growing animals [22] cumulates several sources of error and is less accurate than FCE values that only require measurements of feed intake and live weight to calculate ADG.

## Conclusions

This study in fattening yearling bulls shows that up to 65% of N isotope discrimination between the animal and the diet originated from the digestive processes, particularly in the rumen. Although the overall relationship between Δ^15^N and feed efficiency was strong and significant for all post-absorptive metabolic pools, the within-diet relationship was less consistent and was dependent on the animal pool and type of diet. Notably, rumen-specific pools revealed a contrasted use of N between the two diets. We conclude that in our experimental conditions, digestion processes and the associated microbial activity play an important role in N isotopic discrimination and, consequently, in feed efficiency in fattening yearling bulls. This study also shows that to accurately predict FCE or NUE based on Δ^15^N it is necessary to consider the type of diet. More studies are warranted to elucidate the specific role of rumen microorganisms on the N isotopic discrimination observed in ruminant body tissues.

## Supporting information

S1 Data (XLS)
